# Implementing Early Rehabilitation Strategies for Chronic Obstructive Pulmonary Disease (COPD) Patients Undergoing Mechanical Ventilation in the Intensive Care Unit

**DOI:** 10.7759/cureus.72847

**Published:** 2024-11-01

**Authors:** Sajad A Khwaja, Mohammad A Habib, Rajesh Gupta, Deepika Singla, Ruchi Basista

**Affiliations:** 1 Department of Physiotherapy, Jamia Hamdard, New Delhi, IND; 2 Department of Medicine, Hamdard Institute of Medical Sciences &amp; Research (HIMSR), New Delhi, IND; 3 Department of Medicine, Pentamed Hospital, New Delhi, IND; 4 Department of Physiotherapy, School of Nursing Sciences &amp; Allied Health (SNSAH) - Jamia Hamdard, New Delhi, IND; 5 Department of Physiotherapy, School of Nursing Sciences &amp; Allied Health (SNSAH) - Jamia Hamdard, Delhi, IND

**Keywords:** critical care, early mobilization, physiotherapy, respiratory disorder, ventilator weaning

## Abstract

Background: Patients afflicted with chronic obstructive pulmonary disease (COPD) frequently manifest acute respiratory failure (ARF), characterized by hypercapnia, hypoxia, malnutrition, muscle weakness, heightened work of breathing (WOB), recurrent acute exacerbations, reliance on mechanical ventilation (MV), and difficulties in the weaning phase. Early implementation of rehabilitation interventions holds promise in mitigating prolonged MV and, consequently, reducing intensive care unit (ICU) morbidity and mortality.

Methodology: A prospective study was undertaken involving COPD type 2 respiratory failure patients receiving MV in an ICU setting. Random assignment was employed to allocate patients to either the experimental or control groups. Both groups received chest physiotherapy, range-of-motion exercises, and standard medical and nursing care. The experimental group additionally underwent early active rehabilitation exercises, including limb strength training and progressive mobility tasks. Various parameters such as mechanical ventilator duration, ICU length of stay (LOS), mortality, and occurrence of adverse events were documented. Group differences were analyzed using independent t-tests.

Results: Among 52 patients, 33 were assigned randomly to each group using sealed envelopes. After withdrawals, 15 patients remained in each group. The experimental group had significantly shorter durations of MV (2.29 ± 0.61 vs 2.86 ± 0.66 days; 95% CI: −1.06 to −0.07, t = −2.37, P = 0.02) and ICU stay (7.66 ± 1.17 vs 8.86 ± 1.68 days; 95% CI: −2.28 to −0.11, t = −2.26, P = 0.03) compared to the control group. ICU mortality rates were similar between groups (1.93 ± 0.25 vs 1.93 ± 0.25; 95% CI: −0.19 to 0.19, t = 0.00, P = 1.00). The experimental group had a higher incidence of non-serious adverse events (0.66 ± 0.48 vs 0.26 ± 0.45 events; 95% CI: −0.04 to −0.75, t = 2.31, P = 0.02) and primarily transient physiological changes.

Conclusion: Engaging the early active rehabilitation exercises for mechanically ventilated COPD patients is practical and results in a reduction in MV duration, consequently shortening the ICU LOS.

## Introduction

Chronic obstructive pulmonary disease (COPD) is a significant global health problem associated with increased rates of illness and death [1]. It is identified as persistent inflammation occurring within the airways and lung tissue, leading to increased resistance in the airways and reduced elastic recoil [2]. These pathophysiological changes result in expiratory flow limitation, causing airflow obstruction, reduced lung elasticity, heightened ventilatory demand, and shortened expiratory time. Ultimately, these processes lead to the emergence of auto-positive end-expiratory pressure (PEEP) and dynamic hyperinflation (DH), complicating respiratory function in individuals with COPD [3]. Despite the expiratory nature of these fundamental physiologic changes, compensatory mechanisms come into play through the elevation of inspiratory flow and lung volume. This compensation induces the development of inspiratory muscle fatigue, a key factor centrally involved in the emergence of acute respiratory failure (ARF), described as the acute incapacity to adequately deliver oxygen or remove carbon dioxide from the body, requiring urgent initiation of either invasive or noninvasive ventilation for these patients [4]. In COPD patients who need invasive MV, a significant proportion (45% to 60%) require prolonged periods of ventilator support, which has been linked to adverse effects such as atrophy of diaphragm myofibers and reduced ability of the diaphragm to contract effectively, promoting unrecognized organ failure, referred as ventilator-induced diaphragmatic dysfunction (VIDD) [5,6]. Numerous research studies have highlighted that VIDD contributes to increased rates of in-hospital mortality, occurrences of hospital-acquired pneumonia, oxidative stress, lung tissue hypoxia, prolonged reliance on MV, and higher healthcare costs [7-9]. Additionally, COPD patients on MV are routinely managed by the administration of sedatives, bronchodilators, steroids, and prolonged immobilization, which have been associated with complications including critical illness neuropathies or ICU-acquired weakness, limited joint mobility, pressure ulcers, deep vein thrombosis (DVT), muscle weakness, prolonged period of MV, cognitive impairments, and psychological disturbances [10,11].

The pathophysiological mechanisms underlying muscle weakness at the cellular and molecular level are associated with muscle proteolysis coupled with depressed protein synthesis, disruptions in myofilament organization, damage to the sarcoplasmic reticulum, reduced electrical excitability, and dysfunction of mitochondria [12]. The adverse effects of COPD extend beyond the musculoskeletal system, impacting cardiovascular function and affecting both right and left ventricular diastolic and systolic function [13,14]. The right ventricle (RV) is particularly compromised due to pulmonary parenchymal damage and hypoxia-induced vasoconstriction, leading to elevated pulmonary vascular resistance. Consequently, RV dilation and hypertrophy can occur, potentially shifting the septum toward the left ventricle (LV), thereby impairing LV filling and overall cardiac output [15,16].

Early active rehabilitation exercises are physical activities intended to generate an immediate physiological response that improves respiratory function, cardiovascular efficiency, blood flow, muscle metabolism, and cognitive alertness [17]. These exercises can be commenced within the initial 48 hours in critically ill patients on MV. However, the initiation of early active rehabilitation exercises should be guided by specific criteria, including stable blood flow, adequate blood oxygen levels, and consistent vital signs [18]. In this concern, earlier studies have demonstrated that early active rehabilitation exercises are safe and effective for enhancing muscle strength, consciousness, and quality of life of ICU patients while also reducing complications, shortening MV duration, and decreasing ICU stays, with a low rate of adverse events (<1%) [19-22].

In contrast, a multitude of conflicting viewpoints has been documented in various published studies. Furthermore, the existing studies on early rehabilitation interventions among mechanically ventilated patients exhibit heterogeneity concerning specific inclusion criteria, training methodologies, and outcomes evaluated. Most of these studies encompassed cohorts with diverse disease profiles; not all investigations concentrated on patients with severe respiratory disease with related pulmonary mechanical derangements such as COPD, and a substantial portion did not evaluate outcomes related to weaning. Therefore, a reassessment of the impact of initiating early active rehabilitation exercises for critically ill patients in the ICU is warranted.

In this research investigation, several key elements are implemented that are hypothesized to improve the quality of the collected data. First, the focus is specifically on COPD patients. Second, early active rehabilitation exercises were initiated as soon as the patient was hemodynamically stable. Third, our main outcomes are evaluating MV duration, ICU LOS, mortality rate, and the incidence of adverse events during early active rehabilitation exercises. In light of these factors, our research aims to conduct a comprehensive evaluation of the existing evidence regarding the effects of early active rehabilitation exercises for critically ill patients receiving MV in the ICU.

This article was previously posted on the BMC Pulmonary Medicine preprint server on May 12, 2024.

## Materials and methods

Study design

This research was conducted as a prospective randomized controlled trial (RCT) involving exclusively COPD type 2 respiratory failure patients receiving MV. The study enrolled individuals admitted to the multidisciplinary internal medicine ICU between November 2022 to December 2023. Approval for the trial was acquired from Jamia Hamdard Institutional Ethics Committee, New Delhi, India, on February 23, 2022 (Ref. No.: 02/22(23/02/2022), and the trial was registered in the Clinical Trial Registry of India under CTRI/2022/10/046512 on October 14, 2022. As most participants were unable to provide consent, written informed consent was obtained from the designated "person responsible" for each patient. Patients eligible for inclusion were required to meet the following criteria: individuals aged 30 years and older who were diagnosed with COPD type 2 respiratory failure were prospectively identified and recruited within 24 hours of undergoing mechanical ventilation (MV) via an endotracheal tube in the ICU.

The exclusion criteria were as follows: individuals unable to ambulate independently before their acute ICU illness (excluding the use of canes or walkers), those with a body mass index (BMI) ≥ 45, pre-existing nonverbal cognitive impairment, pre-existing immunocompromised patients, acute stroke, or neuromuscular diseases affecting weaning potential (such as amyotrophic lateral sclerosis, muscular dystrophies, myasthenia gravis, and Guillain-Barré syndrome), pathologic fractures, recent hip fractures, MV for 24 hours before the intervention, cardiopulmonary resuscitation or do-not-resuscitate status at the time of admission, cancer treatment received within the past six months, or hospitalization in 30 days before admission were excluded.

**Figure 1:**
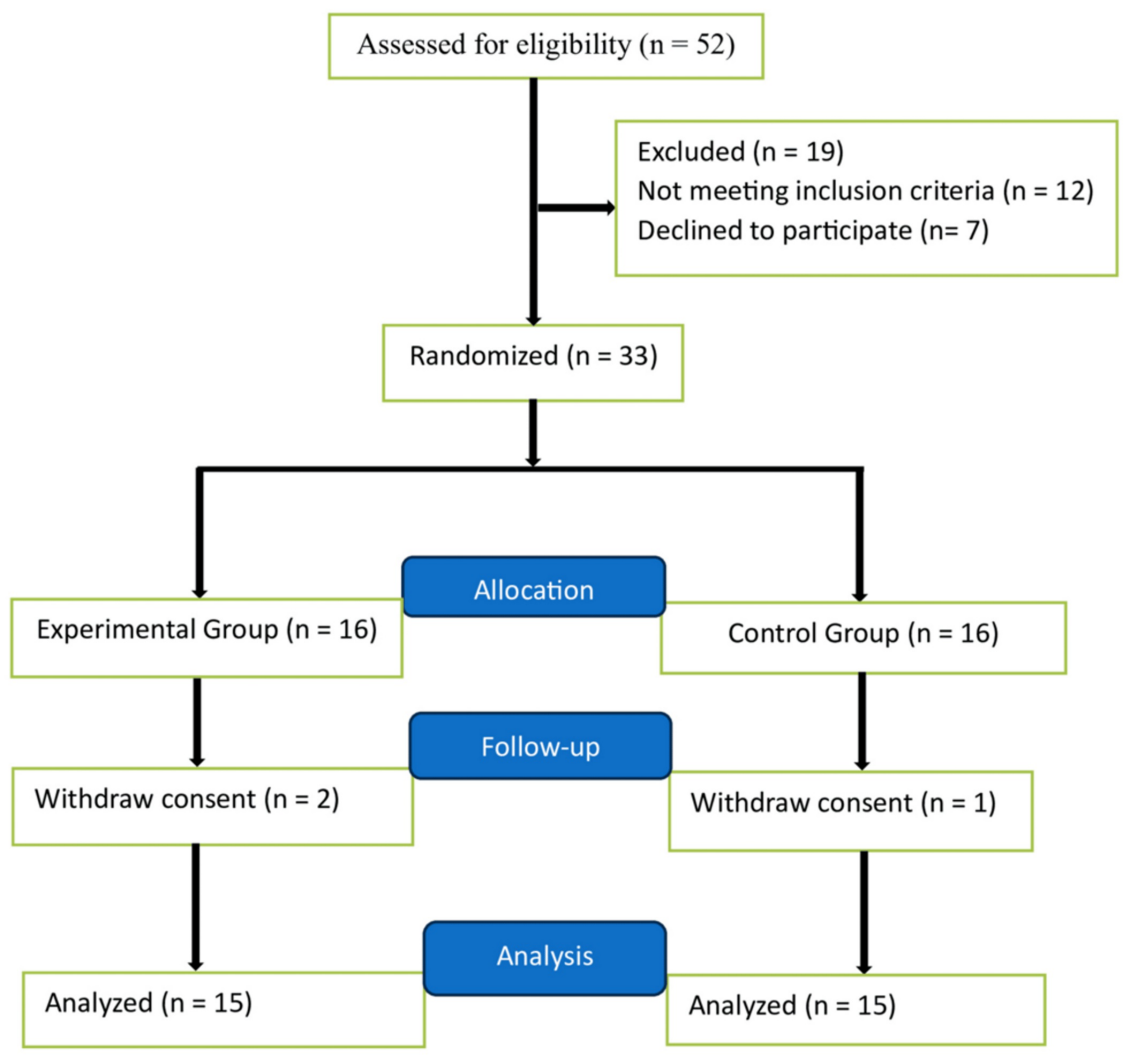
Trial profile n: Number of patients.

The study comprised a cohort of 30 patients who exhibited similar demographics, baseline characteristics, and diagnostic profiles. Patients underwent random allocation into either the experimental or control group using the sealed envelope method for this investigation (Figure 1). All patients of both groups were ventilated via A/C mode with pressure support, until resumption of spontaneous breathing on MV. Maquet ventilators were used in the study. In addition to MV, the protocol for managing patients enrolled in this study includes adequate nutrition support, nebulized bronchodilators, mucolytics, prophylactic anticoagulants, gastric mucosal strengthener, and antibiotics, based on both empirical and sputum culture results. The experimental group underwent regular early active rehabilitation exercise protocol sessions twice daily (morning and evening) led by a team of physiotherapists. These sessions comprised exercises focused on strengthening the limbs and gradual progression to bedside sitting and dangling, then standing, and finally ambulating by the bedside until patients were transferred to the ward. In contrast, the control group received usual care physiotherapy, which involved range-of-motion exercises and positioning the patient in either a supine or lateral decubitus position, along with regular measures like turning, back-clapping, and suctioning. The physiotherapists administering mobilization were not blinded due to the nature of the intervention. However, personnel responsible for assigning and evaluating patient outcomes were unaware of the trial group assignments, and the statistical analysis was conducted in a blinded manner.

Statistical analysis

The statistical analyses conducted on patient data involved the use of IBM SPSS Statistics version 23.0 (IBM Corp., Armonk, NY). Group comparisons were conducted using an unpaired t-test, while the significance of outcome measurements between the two groups was assessed through parametric analysis employing the independent sample t-test. A significance threshold of P-value less than 0.05 was adopted to ascertain the significance of these outcome measurements. The results are depicted as mean values with corresponding standard deviations (mean ± SD).

## Results

During the period from November 2022 to December 2023, initially, 52 patients underwent screening for potential participation in the study. Ultimately, 33 patients who fulfilled the inclusion criteria were randomly allocated to either the experimental group (17 patients) or the control group (16 patients), as depicted in Figure 1 which outlines the trial profile. Of the enrolled participants, consent was subsequently withdrawn by two patients from the experimental group and one patient from the control group, resulting in a final cohort of 15 patients in each group. The baseline demographic and clinical profiles encompassing age, gender, height, weight, BMI, Acute Physiology and Chronic Health Evaluation II (APACHE II) scores, Glasgow Coma Scale (GCS) scores, and duration of COPD were comparable and well-matched between the two groups. Similarly, comprehensive evaluations across both groups were conducted on diagnostic factors such as smoking habits and the presence of comorbidities such as diabetes mellitus (DM), hypertension (HTN), post-tuberculosis history, and post-cardiac artery bypass graft (CABG). Furthermore, an assessment of baseline cardio-respiratory parameters was conducted, which included measurements of blood pressure, arterial blood gas (ABG), renal function tests, potassium levels, hemoglobin, and hematocrit levels. Upon conducting statistical analysis on these baseline parameters, statistical analysis revealed no significant discrepancies among the groups across these baseline factors, as outlined in Table 1.

**Table 1:** Demographic and clinical baseline characteristics of the study groups upon admission H/O: History of; DM: Diabetes mellitus; HTN: Hypertension; TB: Tuberculosis; CABG: Coronary artery bypass graft; D1: First day of patient admission to the intensive care unit (ICU); MAP: Mean arterial pressure; Na: Sodium; K: Potassium; WBC: Weight blood count; GCS: Glasgow Coma Score; pCO_2_: Partial pressure of carbon dioxide; pO_2_: Partial pressure of oxygen; CHCO_3_: Bicarbonate; Hematocrit (Hct c): Percentage of red blood cells; SpO_2_: Oxygen saturation.

Variables	Experimental Group (n = 15)	Control Group (n = 15)	P	t-score
Age (years)	68.60 ± 8.06	69.60 ± 8.58	0.74	−0.32
Sex	0.26 ± 0.45	0.26 ± 0.45	1.00	0.00
Height in cm	161.26 ± 6.38	161.73 ± 7.49	0.85	−0.18
Weight in Kg	66.33 ± 11.36	68.33 ± 10.30	0.61	−0.50
BMI	25.12 ± 3.90	26.17 ± 3.77	0.45	−0.75
Duration of COPD (Years)	10.66 ± 6.11	9.00 ± 4.39	0.39	0.85
Smoking	1.20 ± 0.41	1.06 ± 0.25	0.29	1.05
H/O. DM	1.86 ± 0.35	1.80 ± 0.41	0.63	0.47
H/O. HTN	1.80 ± 0.41	1.73 ± 0.45	0.67	0.41
Past TB	1.93 ± 0.25	1.93 ± 0.25	1.00	0.00
Post CABG	1.93 ± 0.25	1.93 ± 0.25	1.00	0.00
MAP, D1	94.86 ± 15.50	94.80 ± 16.17	0.99	0.01
Serum Na (mmol/L), D1	143.33 ± 5.47	140.40 ± 4.33	0.11	1.62
Serum K (mmol/L), D1	4.53 ± 0.40	4.50 ± 0.57	0.88	0.14
Serum creatinine (umol/L), D1	1.20 ± 0.33	1.14 ± 0.29	0.60	0.51
WBC (total/mm^3^), D1	13166.66 ± 2645.39	12226.66 ± 2692.70	0.34	0.96
GCS, D1	11.20 ± 0.86	11.00 ± 0.84	0.52	0.64
APACHE II score	53.13% ± 10.20%	53.60% ± 12.15%	0.91	−0.11
pH, D1	7.18 ± 0.07	7.18 ± 0.04	0.86	0.17
pCO_2_ in mmHg, D1	108.93 ± 8.58	111.06 ± 11.18	0.56	−0.58
pO_2_ in mmHg, D1	59.53 ± 8.34	60.13 ± 8.84	0.85	−0.19
CHCO_3_ in mmol/L, D1	40.30 ± 6.50	39.97 ± 4.28	0.87	0.16
Hct c, D1	32.46% ± 8.50%	30.13% ±7.50%	0.43	0.79
SpO_2_, D 1	77.56 ± 7.87	77.97 ±7.04	0.88	−0.15
Organism	0.60 ± 1.29	0.40 ± 0.91	0.62	0.48

Outcome measures

Mechanical Ventilation Duration

MV duration was described as the interval from the initiation of MV to the moment when the patient no longer required invasive positive pressure ventilation. This duration included intermittent breaks from MV as part of the weaning process. Patients who remained off MV continuously for at least 24 hours were considered successfully extubated from MV. In the experimental group, the mean MV duration was 5.53 ± 0.74 days, while in the control group, it was 6.20 ± 0.94 days. Analysis between the groups demonstrated a statistically significant difference (95% CI: −1.30 to −0.03, t = −2.15, P = 0.04) in MV duration, as detailed in Table 2.

**Table 2:** Outcome measurements n: Number of patients.

Variables	Experimental Group (n = 15)	Control Group (n = 15)	P	t-score
Mechanical ventilation (MV) duration	5.53 ± 0.74 days	6.20 ± 0.94 days	P = 0.04	−2.15
Post-extubation ICU stay	2.29 ± 0.61 days	2.86 ± 0.66 days	P = 0.02	−2.37
ICU length of stay (LOS)	7.66 ± 1.17 days	8.86 ± 1.68 days	P = 0.03	−2.26
Adverse events	0.66 ± 0.48 events	0.26 ± 0.45 events	P = 0.02	2.31
Mortality	1.93 ± 0.25 events	1.93 ± 0.25 events	P = 1.00	0.00

Post-extubation ICU Stay

The post-extubation ICU stay duration was measured from extubation until the patient's transfer from the ICU to the ward. In the experimental group, the average duration of post-extubation ICU stay was 2.29 ± 0.61 days, compared to 2.86 ± 0.66 days in the control group, as detailed in Table 2. A statistically significant difference among the groups was observed (95% CI: −1.06 to −0.07, t = −2.37, P = 0.02) in the post-extubation ICU stay duration.

ICU Length of Stay

ICU LOS was determined as the duration from ICU admission to the patient's transfer to the ward. In the experimental group, the mean ICU stay was 7.66 ± 1.17 days, compared to 8.86 ± 1.68 days in the control group, as detailed in Table 2. Analysis among the groups revealed a statistically significant difference in the ICU LOS (95% CI: −2.28 to −0.11, t = −2.26, P = 0.03).

Adverse Events

Adverse events were common and primarily included patient-ventilator asynchrony, tachypnea, tachycardia, and desaturation, with patient-ventilator asynchrony being the most common. Notably, no serious adverse events such as desaturation below 80%, unplanned extubations, falls, or fluctuations in systolic blood pressure under 90 mmHg or exceeding 200 mmHg were observed. These events resolved once patients returned to a resting state. The mean number of adverse events in the experimental and control groups was 0.66 ± 0.48 and 0.26 ± 0.45, respectively. A statistically notable disparity was observed in adverse events among the groups (95% CI: −0.04 to −0.75, t = 2.31, P = 0.02) as determined by group analysis, as detailed in Table 2. Overall, while adverse events were generally minor and transient; patient safety measures ensured that no severe complications occurred, allowing for successful management and resolution of these events.

Mortality

In the experimental group, the mean mortality score was 1.93 ± 0.25, which paralleled that of the control group of 1.93 ± 0.25, as detailed in Table 2. So, no statistically notable disparity was observed in mortality events among the groups (95% CI: −0.19 to 0.19, t = 0.00, P = 1.00) based on group analysis. It is noteworthy that the study's relatively small sample size may have affected the ability to detect the potential effects of early active rehabilitation exercises on mortality outcomes.

## Discussion

COPD patients commonly present with ARF, hypercapnia, muscle weakness, recurrent acute exacerbations, dependence on MV, and challenges in the weaning process [23]. These complications collectively highlight COPD as a significant global health challenge marked by elevated rates of morbidity and mortality [1,24]. Aligned with prior research [19-22,25], our study affirms the viability and safety of early mobilization in intubated ICU patients. This RCT encompassing COPD patients undergoing MV revealed that early active rehabilitation exercises were linked with a statistically significant reduction in ICU LOS (95% CI: −2.28 to −0.11, t = −2.26, P = 0.03) and fewer days on MV (95% CI: −1.30 to −0.03, t = −2.15, P = 0.04). However, it was accompanied by an increase in adverse events (95% CI: −0.04 to −0.75, t = 2.31, P = 0.02), predominantly transient physiological changes such as patient-ventilator asynchrony, elevated heart rate, and desaturation, none of which necessitated serious medical intervention. The comparison of ICU mortality risks among the experimental and control groups indicated that early active rehabilitation exercises did not demonstrate a statistically significant impact, and both groups exhibited a similar ICU mortality rate (95% CI: −0.19 to 0.19, t = 0.00, P = 1.00).

Our study's results contrast with those of the RCT published by Hodgson et al. [25]. It is important to acknowledge that we did not include patients with diverse disease profiles or ICU types, as these factors could influence the results. Notably, an increase in hospital mortality rates has been observed among surgical patients in the surgical ICU, highlighting the need for careful adjustments to mobilization protocols based on patient wounds and illness. Improper implementation of mobilization therapy can result in negative outcomes or mortality. Additionally, the reduction in LOS in the ICU and hospital has a more pronounced impact on patients without respiratory diseases compared to those with respiratory diseases in the respiratory care unit. These varying factors contribute to disparate outcomes across trials, making direct comparisons challenging. Consequently, our study exclusively recruited patients diagnosed with COPD respiratory type 2 failure.

Our study findings align with a recent meta-analysis conducted by Wang et al. [26], which indicated that early mobilization resulted in a reduction in ICU LOS (mean difference: −2.18 days, 95% CI: −4.22 to −0.13, P = 0.04), a decrease in MV duration (mean difference: −2.27 days, 95% CI: −3.99 to −0.56, P = 0.009), and an increase in the adverse events among early mobilization patients compared to the standard mobilization protocols group (risk ratio: 1.99, 95% CI: 1.25 to 3.16, P = 0.004). Additionally, our findings are consistent with Lai et al.'s observational study [27], which demonstrated a significant reduction in MV duration (4.7 days compared to 7.5 days) and a decreased ICU LOS (6.9 days compared to 9.9 days) among patients with ARF.

Previous meta-analyses and studies, including Zhang et al. [28], reported similar findings, demonstrating that early mobilization significantly reduces the risk of ICU-acquired weakness by 40% to 90% among critically ill patients in ICU (P = 0.01). This intervention also improved functional capacity, with a higher proportion of patients able to stand (90% vs. 62%, P = 0.02) and increased ventilator-free days (mean difference: 0.17, 95% CI: 0.02, 0.31; P = 0.02). Patients who underwent early mobilization also showed improved unassisted walking ability at hospital discharge (average 33.4 meters (with a range from 0 to 91.4 meters) vs. 0 meters (ranging from 0 to 30.4 meters), P = 0.00), and a 16% higher probability of being discharged directly to home (P = 0.04, 95% CI: 1.00, 1.34). However, there was a non-significant increase in mortality and adverse event rates associated with early mobilization.

Escalon et al. [29] demonstrated significant improvements following the implementation of an early mobilization program in 2015 for patients on prolonged MV across five ICUs compared to 2014. The mean ICU LOS decreased from 34.40 (SD = 21.87) to 30.57 days (SD = 22.4), while the overall hospital LOS declined from 52.70 (SD = 35.48) to 43.30 days (SD = 30.61). Excess hospital days were reduced from 16.51 (SD = 35.22) to 6.47 days (SD = 30.6). Additionally, the time to initiate physical therapy improved from 20.09 (SD = 14.06) to 14.78 days (SD = 12.12), and the average number of physical therapy follow-up consultations increased from 6.14 (SD = 5.21) to 7.73 sessions (SD = 7.93).

Schweickert et al. [20] reported a comprehensive whole-body rehabilitation strategy, involving early sedation interruption and implementation of early rehabilitation on critically ill undergoing MV, which was found to be safe and well-received. This approach resulted in improved functional outcomes upon hospital discharge for 29 (59%) patients compared to 19 (35%) patients receiving standard care. Additionally, patients who underwent the rehabilitation approach had reduced duration of delirium (median: 2.0 days, IQR: 0.0-6.0) compared to those receiving standard care (median: 4.0 days, IQR: 2.0-8.0; P = 0·02) and a greater duration without ventilator support (median: 23.5 days, IQR: 7.4-25.6 compared to median: 21.1 days, IQR: 0.0-23.8, P = 0.05).

## Conclusions

COPD patients commonly develop ARF characterized by muscle weakness, increased work of breathing (WOB), frequent exacerbations, dependence on MV, and challenges in the weaning process. In conclusion, the current study indicates that early active rehabilitation exercises were effective in reducing duration of MV and shortening ICU stays. Given the potential constraints of our study, additional large-scale, meticulously conducted RCTs are required to confirm our observations.

## Appendices

**Table 3:** Patient data utilized for statistical analysis Group: (Experimental group: 1, Control group: 2). Sex: (Male: 0, Female: 1). BMI: Body Mass Index; COPD: Chronic Obstructive Pulmonary Disease. Smoking status: (Yes: 1, No: 0). History of diabetes mellitus (H/O DM): (Yes: 1, No: 2). History of hypertension (H/O HTN): (Yes: 1, No: 2). History of tuberculosis (Past TB): (Yes: 1, No: 2). History of coronary artery bypass grafting (Post-CABG): (Yes: 1, No: 2). MAP: Mean arterial pressure. D1: First day of patient admission to the intensive care unit (ICU). Na: Sodium. K: Potassium. WBC: White blood cell count. Organisms: (E. coli: 1, *Pseudomonas aeruginosa*: 2, *Streptococcus pneumoniae*: 3, and *Klebsiella*: 4). MV: Mechanical ventilation. Number of days in ICU post-extubation: (*: No patient). Adverse events: (Yes: 1, No: 0). Motility: (Yes: 1, No: 2).

Group	Age (Years)	Sex	Height in cm	Weight in Kg	BMI	COPD duration (Years)	Smoking	H/O DM	H/O HTN	Past TB	Post CABG	Temperature in C, D1	MAP, D1	Serum Na (mmol/L), D1	Serum K (mmol/L), D1	Serum creatinine (umol/L), D1	WBC (total/mm^3^), D1	Glasgow Coma Score (GCS), D1	APACHE II Score	pH, D1	pCO_2_ in mmHg, D1	po_2_ in mmHg, D1	cHCO_3_ in mmol/L, D1	Hct c, D1	Sp O_2_, D1	Organism	No. of days on MV	No. of days in ICU post extubation	Total no. of days in ICU	Adverse events	Motility
1	60	0	166	56	20.322	6	1	2	2	2	2	38.8	90	133	4.3	0.8	10600	11	55%	7.2	113	63	42.7	26%	95.2	0	5	2	7	1	2
1	79	0	164	59	21.936	5	1	2	2	2	2	37.7	103	137	4.6	1	8600	11	55%	7.08	105	52	30.7	42%	67.3	0	6	3	9	0	2
1	70	1	156	64	26.298	5	1	2	2	2	2	37.2	93	152	5.1	1.6	16200	11	55%	7.09	128	65	37.2	35%	86.8	0	5	1	6	1	2
1	51	0	162	82	31.245	18	1	1	1	2	2	36.1	57	139	4.5	1	12500	11	24%	7.25	100	56	39.5	38%	82.2	2	7	2	9	1	2
1	80	1	153	48	20.584	6	2	2	2	2	2	37.7	80	141	4	0.9	15000	11	55%	7.18	98	48	37.9	21%	78.5	0	5	2	7	0	2
1	68	0	161	72	27.776	12	1	2	2	2	2	38.3	110	142	4.5	1.1	11300	13	55%	7.3	105	67	49.9	23%	84.3	4	5	2	7	1	2
1	65	0	155	58	24.141	9	2	2	2	2	2	39.4	97	144	5	1.5	13100	11	55%	7.15	110	55	37	42%	75.6	3	5	3	8	1	2
1	58	0	153	64	24.386	15	1	2	2	1	2	37.2	83	150	4.6	1.1	12700	13	40%	7.28	98	58	46.5	39%	81.7	0	6	*	6	0	1
1	67	1	160	84	32.812	16	1	2	2	2	2	38.8	103	141	4	1.3	13800	11	55%	7.12	115	61	33.6	22%	71.3	0	5	3	8	1	2
1	75	0	165	57	20.936	16	1	2	1	2	1	36.6	107	139	5.1	0.7	15200	11	55%	7.2	105	44	39.7	37%	77.1	0	5	2	7	1	2
1	77	0	157	58	23.53	4	1	1	2	2	2	37.7	80	151	4.8	1.6	10600	11	73%	7.32	110	57	52.2	40%	79.4	0	7	3	10	1	2
1	69	1	160	67	26.171	8	1	2	2	2	2	38.8	90	147	4.3	1.8	18300	11	55%	7.15	115	75	38.9	42%	72.8	0	6	2	8	1	2
1	71	0	165	64	21.049	25	1	2	1	2	2	38.3	117	145	4.9	1.5	14100	10	55%	7.1	100	58	30	21%	69.2	0	5	2	7	0	2
1	74	0	164	76	28.256	9	1	2	2	2	2	36.6	103	141	3.8	0.9	10000	10	55%	7.22	112	62	42.3	35%	76.3	0	6	3	9	0	2
1	65	0	178	86	27.43	6	2	2	2	2	2	38.8	110	148	4.5	1.2	15500	12	55%	7.18	120	72	46.4	24%	65.7	0	5	2	7	1	2
2	64	0	167	74	26.533	8	1	2	2	2	2	37.2C	110	139	4.5	1.1	9200	11	55%	7.18	100	58	33.6	42%	78.8	0	5	2	7	0	2
2	62	0	164	65	24.167	23	1	1	2	2	2	39.4C	73	142	4.3	0.9	12300	10	73%	7.25	120	45	49.6	23%	83.2	0	6	3	9	1	2
2	68	1	157	68	27.587	11	1	2	2	2	2	37.7C	83	138	3.8	1.3	9800	10	55%	7.23	125	67	47.2	21%	76.6	1	7	3	10	0	2
2	71	1	154	57	24.034	6	1	2	2	2	2	38.3C	67	134	5.1	0.7	14200	10	55%	7.15	110	76	36.9	28%	79.7	0	7	4	11	1	2
2	68	0	163	90	33.874	9	1	1	1	2	2	38.8C	117	136	3.8	0.8	10800	11	55%	7.16	120	58	40.4	42%	83.5	0	8	3	11	0	2
2	58	0	159	69	27.293	8	1	1	2	2	2	37.7C	90	148	4.5	1.3	13200	13	40%	7.2	105	58	39.7	35%	81.4	0	6	2	8	0	2
2	76	0	171	57	19.493	7	1	2	2	1	2	37.7C	107	143	4.8	1.7	15300	11	73%	7.12	125	53	38.7	21%	78.3	0	5	3	8	0	2
2	84	1	159	60	23.733	8	1	2	2	2	2	38.8C	113	137	3.9	1.1	18400	11	73%	7.21	115	57	43.5	26%	74.3	0	5	*	5	0	1
2	54	0	147	67	31.005	12	2	2	2	2	2	37.2C	113	144	5.3	1.4	11800	11	40%	7.2	95	62	35.8	25%	68.8	0	7	3	10	1	2
2	72	0	164	66	24.538	10	1	2	1	2	2	36.6C	83	141	4.7	1.2	13800	12	40%	7.25	98	65	41.6	24%	83.3	2	7	2	9	0	2
2	76	0	174	91	30.056	9	1	2	1	2	2	37.2C	93	143	5.2	0.7	10400	11	40%	7.15	115	47	38.9	42%	92.8	0	6	3	9	0	2
2	66	0	168	66	23.384	6	1	2	2	2	1	37.7C	83	136	4.6	0.9	7800	11	40%	7.1	128	76	38.4	35%	82.5	0	7	4	11	0	2
2	75	1	150	64	28.444	4	1	2	2	2	2	38.8C	103	148	5.3	1.4	11700	12	55%	7.14	105	64	37.6	32%	68.3	0	6	3	9	1	2
2	66	0	163	72	27.099	6	1	2	2	2	2	37.7C	107	136	4.2	1.1	14100	10	55%	7.22	95	55	36	29%	68.3	0	5	2	7	0	2
2	84	0	166	59	21.41	8	1	2	1	2	2	36.6C	80	141	3.6	1.5	10600	11	55%	7.2	110	61	41.7	27%	69.8	3	6	3	9	0	2
